# The mitogenome of a medically important paper wasp, *Polistes hebraeus* (Hymenoptera, Vespidae)

**DOI:** 10.1080/23802359.2022.2087560

**Published:** 2022-06-24

**Authors:** Xi Feng, Sai Hu

**Affiliations:** a Affiliated Changsha Hospital of Hunan Normal University, Changsha, Hunan, China; b The Fourth Hospital of Changsha, Changsha, Hunan, China; c Institute of Emergency and Critical Care Medicine of Changsha, Changsha, Hunan, China

**Keywords:** Mitogenome, Vespidae, venom, sting, phylogenetic

## Abstract

*Polistes hebraeus* (Smith) is a common paper wasp species of subfamily Polistinae. The clinical potential allergic reaction after stings and valuable venom components of *P. hebraeus* make it a medical importance species. Here, the mitochondrial genome of *P. hebraeus* was analyzed. The mitogenome was 19,262 bp in length including 22 transfer RNAs (tRNA) and 2 ribosomal RNAs (rRNA). The protein coding gene (PCGs) number and order are consistent with existing records of Polistidae. The nucleotide composition is AT: 84.5% and CG: 15.5% which are largely similar with other complete Polistidae mitogenome (15.27 ∼ 19.16%). The available mitogenome resources of Polistidae species and *P*. *hebraeus* were used to construct the phylogenetic tree. It showed that the genus *Polistes* could clearly seperated from *Parapolybia*, and the relationship in *Polistes* was consistent with previous studies. This mitogenome resource can contribute to further phylogenetic and taxonomic study on paper wasp.

Sting of wasps could cause clinical syndromes like itching, swelling and acute pain, or even serious outcomes. Clinical case reports of wasp stings caused serious allergic reactions are common in emergency department of hospitals globally (Bilo and Bonifazi 2008). Species of *Polistes* were commonly called paper wasp which could construct nests using papery materials. *Polistes hebraeus* (Fabricius, 1787) (Hymenoptera, Vespidae) is a middle size paper wasp wildly distributed in Southeast countries of China (Sinica ECoFSA 1985). The habitat of *P. hebraeus* and some close species are usually overlapped with human habitation areas, which brings risks to residents living around. Venom components of *P. hebraeus* have been analyzed previously for its medicinal consideration (Mortari et al. 2005; Savi et al. 2016). Wasps like *P. hebraeus* are commonly used as traditional medicine in China (Liang et al. 1994). For the potentially medical importance of *P. hebraeus*, a complete mitogenome (GenBank: OM423599) was analyzed and provided here.

The *P. hebraeus* were captured from Liuli Mountain, Nanning City, Guangxi Province, China (108°50′N, 22°76′E). The wasps were found building a nest under the roof of a local house and collected using a nylon insect net at night. The samples were deposited into pure ethyl alcohol.

The voucher specimens of *P. hebraeus* were restored at the bio-sample herbarium of Institute of Emergency and Critical Care Medicine of Changsha and recorded with a unique series code (MG202201-1) (http://www.cs4hospital.com/, Xi Feng, csssyyfengxi@sina.com).

Total genomic DNA was extracted from the thorax part of wasp using a modified cetyltrime thylammonium ammonium bromide (CTAB) method and applied to 500 bp paired-end library construction using the NEBNext Ultra DNA Library Prep Kit for Illumina sequencing. Sequencing was carried out on the Illumina NovaSeq 6000 platform (BIOZERON Co., Ltd, Shanghai, China). Approximately 5 Gb of raw data was generated with 150 bp paired-end read lengths. Ethical approval permission is not applicable for none animals were used in present work.

*De novo* assembly with GetOrganelle v1.6.4 produced contigs of mt genome (Jin et al. 2020). A number of potential mitochondrial reads were extracted from the pool of Illumina reads using BLAST searches against mt genomes of related species *Polistes riparius*-ref (LC519884) and the GetOrganelle result. The mitochondrial Illumina reads were obtained to perform *de novo* assembly using the SPAdes-3.13.1 package (Bankevich et al. 2012). The GetOrganelle assembly contigs were optimized by scaffolds from SPAdes result. Finally, the assembled sequences were reordered and oriented according to the reference mt genome,

The mitochondrion genes were annotated using the online MITOS2 tool (Bernt et al. 2013) with default parameters to predict protein coding genes, transfer RNA (tRNA) genes, and ribosome RNA (rRNA) genes. The position of each coding gene was determined using BLAST search against *P. riparius*-ref mt genes. Manual corrections of genes for start/stop codons were performed in SnapGene Viewer (from Insightful Science; available at snapgene.com) by referencing the *P. riparius* mt genome. The circular mt genome map of *P. hebraeus* was drawn using the CGview tool (Grant et al. 2012). Functional annotations were performed using sequence-similarity Blast search with a typical cutoff E-value of 10^−5^ against several publicly available protein databases: NCBI non-redundant (Nr) protein database, Swiss-Prot, Clusters of Orthologous Groups (COGs), and Kyoto Encyclopedia of Genes and Genomes (KEGG) and Gene Ontology (GO) terms.

Clean reads were finally assembled into a complete circular mitochondrial genome of 19,262 bp in length. The nucleotide composition was AT: 84.5% and CG: 15.5% (T:42.11%, A:42.42%, C:10.15%, G:5.32%), which are largely similar with other complete Polistidae mitogenome. In total, 13 PCGs, 22 tRNA, and 2 rRNA genes were annotated. The PCGs number and order is consistent with existing records of Polistidae. For phylogenetic analysis, a Maximum Likelihood tree was constructed based on 13 PCGs genes of *P. hebraeus* and the available sequences in Polistinae using MEGA 7 software with bootstrap of 1000 replicates (Kumar et al. 2016). *Antodynerus aff. limbatus* and *Orancistrocerus aterrimus* was set as outgroups (Figure 1). The result showed that all the species were well divided with high supporting value. The genus *Polistes* could clearly seperated from *Parapolybia*, and the relationship under the *Polistes* was consistent with previous studies (Piekarski et al. 2018; Yamasaki et al. 2020). *Polistes hebraeus* is very close to *Polistes jokahamae*. This mitogenome resource can contribute to further phylogenetic and taxonomic study on paper wasps.

**Figure 1.**
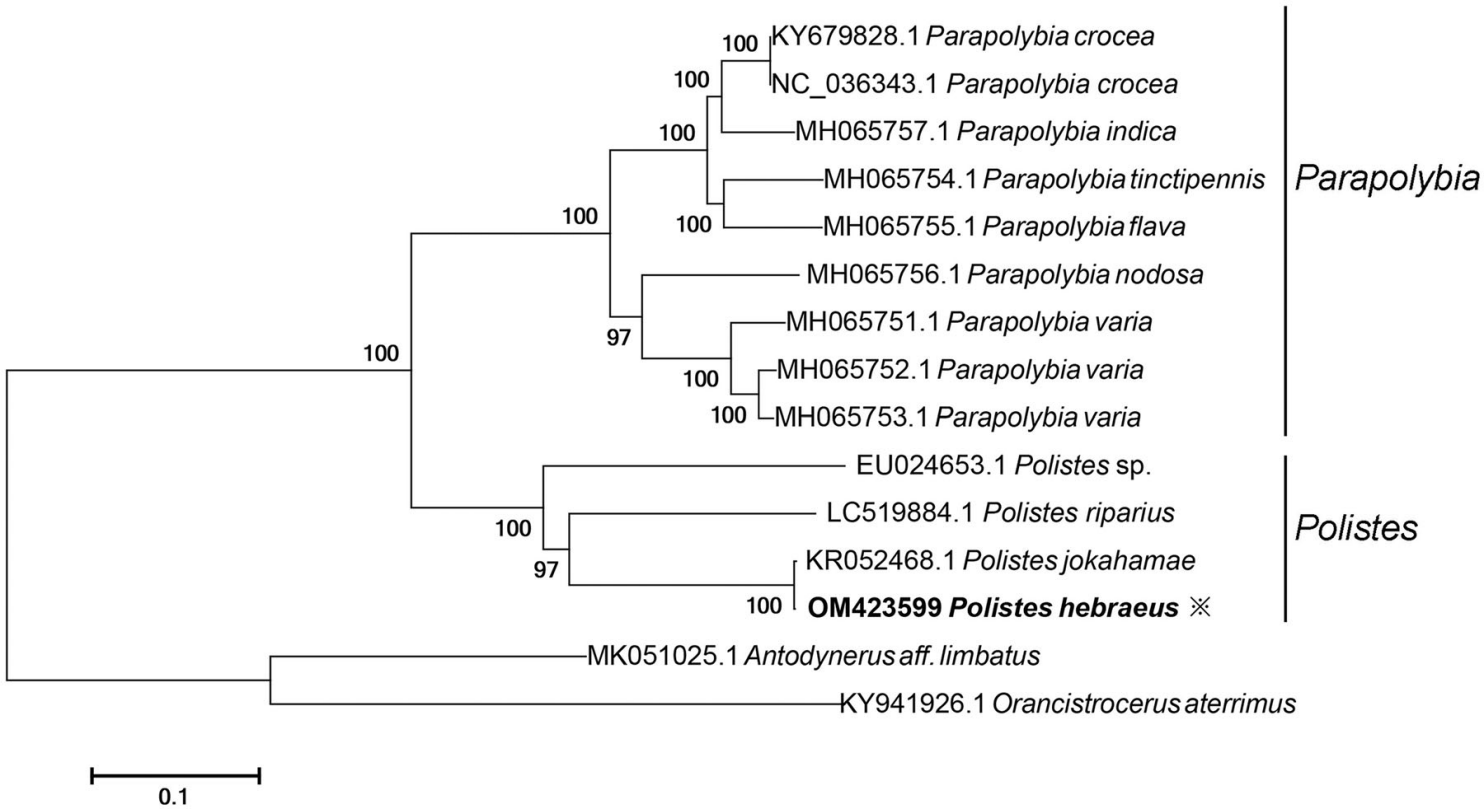
The phylogenetic tree was constructed based on *P. hebraeus* and the available sequences in Polistinae using MEGA 7 software (bootstrap = 1000 replicates).

## Ethical approval

No ethical approval is required in present work.

## Author contributions

Sai Hu supervised and supported this work, and finally confirmed the approval of the version. Xi Feng designed and performed the work, analyzed the data and prepared the paper. All authors agree to be accountable for all aspects of the work.

## Data Availability

The genome sequence data that support the findings of this study are openly available in GenBank of NCBI at [https://www.ncbi.nlm.nih.gov] under the accession no. OM423599. The associated BioProject, SRA, and Bio-Sample numbers are PRJNA801775, SRS11816958, SAMN25385376 respectively.
